# Is SARS‐CoV‐2 Infection (COVID‐19) Associated With Congenital Anomalies? A Population Study of Congenital Anomaly Surveillance Data and Maternal SARS‐CoV‐2 Infection in Wales

**DOI:** 10.1002/bdr2.70122

**Published:** 2026-10-30

**Authors:** Samantha Rose Fisher, Robert Maddison‐Jarvis, Penelope Cresswell‐Jones, David Tucker, Llion Davies

**Affiliations:** ^1^ Congenital Anomaly Register and Information Service Public Health Wales Cardiff UK; ^2^ Wales Gene Park Cardiff University Cardiff UK; ^3^ Swansea Bay University Health Board Port Talbot UK

**Keywords:** congenital anomalies, COVID‐19, disease registries, pregnancy, surveillance

## Abstract

**Objectives:**

Explore the relationship between maternal SARS‐CoV‐2 infection and congenital anomalies in Wales.

**Study Design:**

Mixed observational epidemiological cross‐sectional and cohort.

**Methods:**

Congenital anomaly prevalence data were provided by the Congenital Anomaly Register and Information Service. Two comparisons were made using these data: firstly, congenital anomaly subgroup prevalence prior to and during the pandemic at population level, and in a cohort of women with laboratory‐confirmed maternal SARS‐CoV‐2 infection compared with the population trend.

**Results:**

Prevalence of congenital anomalies in Wales was lower during the pandemic (mean 365 per 10,000 in 2020–22 vs. 454 per 10,000 in 2009–19) showing stable trends. However, the mean for abdominal wall defects increased from 9.8 per 10,000 births between 2009 and 2019 to 13.9 per 10,000 births in 2020–2022, largely attributed to an increase in omphalocele. A cohort of 486 confirmed maternal SARS‐CoV‐2 infections led to 181 births affected by congenital anomalies, and the trimester of infection did not impact the adjusted odds of this occurring. Circulatory anomalies were most frequently reported, consistent with pre‐pandemic population‐level data. Urinary and cardiac anomalies were more prevalent in this cohort group than at population level.

**Conclusions:**

There was no clear evidence at population‐level of an association between congenital anomalies and maternal SARS‐CoV‐2 infection in Wales. However, a potential association with specific congenital anomalies cannot be excluded. Increased omphalocele is consistent with a Europe‐wide trend that pre‐dated COVID‐19. National surveillance registries should remain vigilant.

## 1 Introduction

The Congenital Anomaly Register and Information Service for Wales (CARIS) aims to provide reliable data on congenital anomalies in Wales, and to assess patterns of anomalies, possible clusters and their causes to inform health services (CARIS 2025). CARIS is part of a European network of population‐based registries named EUROCAT, which have a role in epidemiological research, surveillance of congenital anomalies, improvement of healthcare and public health policy support across most European countries (European Commission, n.d.‐a). An important function of congenital anomaly surveillance is early identification of teratogenic threats.

Maternal infections have been known teratogens for several decades. Studies as early as 1966 linked maternal rubella and cytomegalovirus infections with the development of the fetus due to their metabolic processes and placental disruption (Wright and Angeles, n.d.). Subsequent research has implicated maternal Herpes simplex 1–2, Herpes virus 6, Varicella zoster, Hepatitis B, Hepatitis C, Parvovirus B19, non‐poli Enteroviruses, influenza, and Zika virus with congenital anomalies (Auriti et al. 2021; Mátrai et al. 2022; Centers for Disease Control and Prevention (CDC) 2023; Fisher et al. 2026). The World Health Organization (WHO) confirmed there were scientific links between the Zika virus and Guillain‐Barré syndrome, microcephaly, and other congenital brain abnormalities (Krauer et al. 2017).

Evidence to date suggests that the overall prevalence of congenital anomalies has not increased above baseline levels following the COVID‐19 pandemic (Calvert et al. 2023; Hernández‐Díaz et al. 2022), although the quality of evidence is variable. A study in Canada observed an increase in microcephaly during the later stages of the COVID‐19 pandemic but suggested there may be observer bias in the confirmation of cases (Auger et al. 2024). Another small cohort study from the United States of America recorded higher rates of microcephaly but confirmed the limitations of diagnosing microcephaly and its difference to small gestational growth, which has already been linked to SARS‐CoV‐2 infection in pregnancy (Moriarty et al. 2025). Increased prevalence of microcephaly has not been observed globally, with studies in India and a large study in the Democratic Republic of Congo specifically highlighting no increase in microcephaly during the pandemic period (Goyal et al. 2024; Arena et al. 2023). A study based in China using live birth data from over 26 million births in the United States of America showed no increase in congenital anomalies except for a peak in cardiac anomalies in the initial pandemic period (Chen and Wang 2025). The increased incidence in congenital heart disease was also observed in China; however, it has been suggested that this could possibly be attributed to the reallocation of medical resources to prioritize cardiology and deprioritize other areas of care in response to the pandemic (Qu et al. 2025).

In Europe, the prevalence of congenital anomalies was 265.24 per 10,000 births between 2005 and 2023, and this has not risen to unprecedented levels since the start of the COVID‐19 pandemic (European Commission, n.d.‐b). Approaches to exploring the impact of COVID‐19 (SARS‐CoV‐2) on congenital anomalies have included comparing the pre‐pandemic to pandemic incidence (Heidarzadeh et al. 2022; Neelam et al. 2023; Reppucci et al. 2023; Shafie et al. 2023) and comparing pregnant women with SARS‐CoV‐2 infection to pregnant women without SARS‐CoV‐2 infection (Cosma et al. 2021, 2022; Crovetto et al. 2021; Eltemamy et al. 2021; Hernández‐Díaz et al. 2022; Hughes et al. 2023; Sabbagh et al. 2023; Kumarasinghe et al., n.d.). The first trimester of pregnancy is particularly important to fetal structural development, with some studies analyzing first trimester infection, but these typically had small sample sizes (Shafie et al. 2023; Cosma et al. 2021, 2022; Eltemamy et al. 2021; Hughes et al. 2023; Meng et al. 2022).

Overall, these studies identified no general association with SARS‐CoV‐2 infection in pregnancy and congenital anomalies. Whilst it is reassuring that there is no clear sign of adverse fetal outcomes from maternal SARS‐CoV‐2 infection generally, it is likely that the true impact of COVID‐19 on fetal development remains unclear due to inadequate infection recording and methodological flaws in published studies (Samara et al. 2024). Studies to date have been limited by factors including sample size, timing, and generalizability.

Population level congenital anomaly registers provide an opportunity to contribute to this evidence base, to help build consensus on the overall effect of COVID‐19 on congenital anomalies (Khan et al. 2020) and allow us to remain vigilant as more evidence emerges. In early 2020, CARIS began capturing data on SARS‐CoV‐2 infections in pregnancy for surveillance purposes as part of the Public Health Wales NHS Trust pandemic response and to establish an analysis cohort. However, as case numbers rapidly increased, data collection was limited to only those reporting congenital anomalies. Evaluating prevalence of congenital anomalies in this cohort presents the opportunity to contribute to this evidence base.

The aim of this study was to explore the relationship between maternal SARS‐CoV‐2 infection and the incidence of congenital anomalies using national population‐level congenital anomaly surveillance data from Wales.

## 2 Methods

### 2.1 Data Sources

Case numbers of congenital anomalies in Wales were obtained from CARIS. CARIS holds data on congenital anomalies from 1998; however, demographics, environment, technology, and maternal age have changed over time (Braz et al. 2018; Mamasoula et al. 2023; Monier et al. 2023; Naidoo and Lakhoo 2025; Shad et al. 2026; Venkatachalam and Siddique 2025). Therefore, data from 2009 onwards were used for this study to provide a more contemporary baseline for these analyses. Anomalies were categorized using the WHO ICD‐10 version 2016 (World Health Organization 2016) section XVII for “Congenital malformations, deformations, and chromosomal abnormalities and pre‐defined CARIS and EUROCAT definitions” (CARIS 2025; European Commission 2025).

Congenital anomalies were grouped into subgroups by organ system because of shared etiological mechanisms or clinical characteristics as per EUROCAT guidance matching CARIS and EUROCAT for comparability (Bergman et al. 2024). Sensorineural hearing loss was added as a subgroup although it is outside chapter Q of ICD‐10 to correspond with published CARIS data. Abdominal wall defects are not included in the gastrointestinal subgroup as these are musculoskeletal anomalies rather than gastrointestinal tract anomalies. We analyzed 11 congenital anomaly subgroups that were directly comparable and covering the main organ systems.

### 2.2 Analysis

This is a mixed retrospective study combining cross‐sectional and cohort study designs. Two complementary analytical approaches were used to distinguish between population‐level temporal changes and individual‐level risk. First, the prevalence of each subgroup during the pandemic years (2020–2022) was compared to the prevalence in the years preceding the COVID‐19 pandemic (2009–2019) at population‐level to assess trends using official statistics for congenital anomalies in Wales published by CARIS. Second, a cohort analysis comparing mothers with SARS‐CoV‐2 infection during pregnancy to the contemporaneous population (years 2020–2022) was conducted to evaluate whether infection was associated with any additional risk beyond the background pandemic‐period prevalence.

### 2.3 Pre‐Pandemic and Pandemic Comparison of Population‐Level Data (2009–2019 vs. 2020–2022)

To understand population‐level associations between congenital anomalies and the pandemic period, the number of congenital anomalies by subgroup and the rate per 10,000 live births were compared between pre‐pandemic (2009–2019) and pandemic (2020–2022) periods. The total cases for each anomaly subgroup and total number of births in Wales (Office for National Statistics 2025; National Community Child Health Database 2025) for each period were used to calculate a prevalence per 10,000 births. A one‐tailed two‐proportion *z*‐test with Benjamini–Hochberg correction to control the false discovery rate (FDR) in multiple hypothesis testing (National Community Child Health Database 2025) for each subgroup was completed to test the null hypothesis that maternal SARS‐CoV‐2 infection did not increase the prevalence of congenital anomalies in Wales (Chen et al. 2017).

Congenital anomaly registries such as CARIS rely heavily upon data being shared to capture cases. Data provision often has a lag time, meaning that there is an expected lag in reporting that will have a greater impact on the most recent years of reporting, and increased pandemic‐related workload on clinicians may have exacerbated this. Lag time varies by congenital anomaly type as some are diagnosed later in the child's life. For this reason, statistical analysis was carried out using a one‐tailed test to exclusively monitor a potential increase in congenital anomaly prevalence. A two‐tailed test risked identifying a reduction in prevalence due to reporting lag.

### 2.4 SARS‐CoV‐2 Infection Cohort Data

To understand the potential association of SARS‐CoV‐2 maternal infection with congenital anomalies, prevalence of congenital anomaly subgroups in a cohort of women with laboratory‐confirmed SARS‐CoV‐2 infection during pregnancy was compared with the population level. The cohort mostly comprised pregnancies where congenital anomalies were suspected and was assembled by the following process, as visualized in Figure 1. All cases of women who tested positive for SARS‐CoV‐2 during pregnancy in Wales were identified by cross‐referencing cases reported to CARIS with laboratory testing data to confirm infection. Women registered with CARIS were identified and linked to laboratory data to identify cases positive for SARS‐CoV‐2 during pregnancy and added to the cohort. All other lab confirmed cases were excluded as the large number was unsustainable to record at the population level; therefore, this should be seen as a convenience sample and not a population‐based cohort. Cases were then assessed against pre‐defined inclusion and exclusion criteria. To be included, cases had to be Welsh residents (assessed using registered postcode). Exclusion criteria were SARS‐CoV‐2 test performed after pregnancy ended; gestation when positive for SARS‐CoV‐2 not known; end date of pregnancy not known; no lab confirmed SARS‐CoV‐2 infection; and incomplete data on anomalies and outcomes. Chromosomal anomalies were excluded as they are commonly caused by genetic rather than environmental factors (World Health Organization n.d.‐b). Pregnancies ending in termination or stillbirth were included where a congenital anomaly outcome was reported.

**FIGURE 1:**
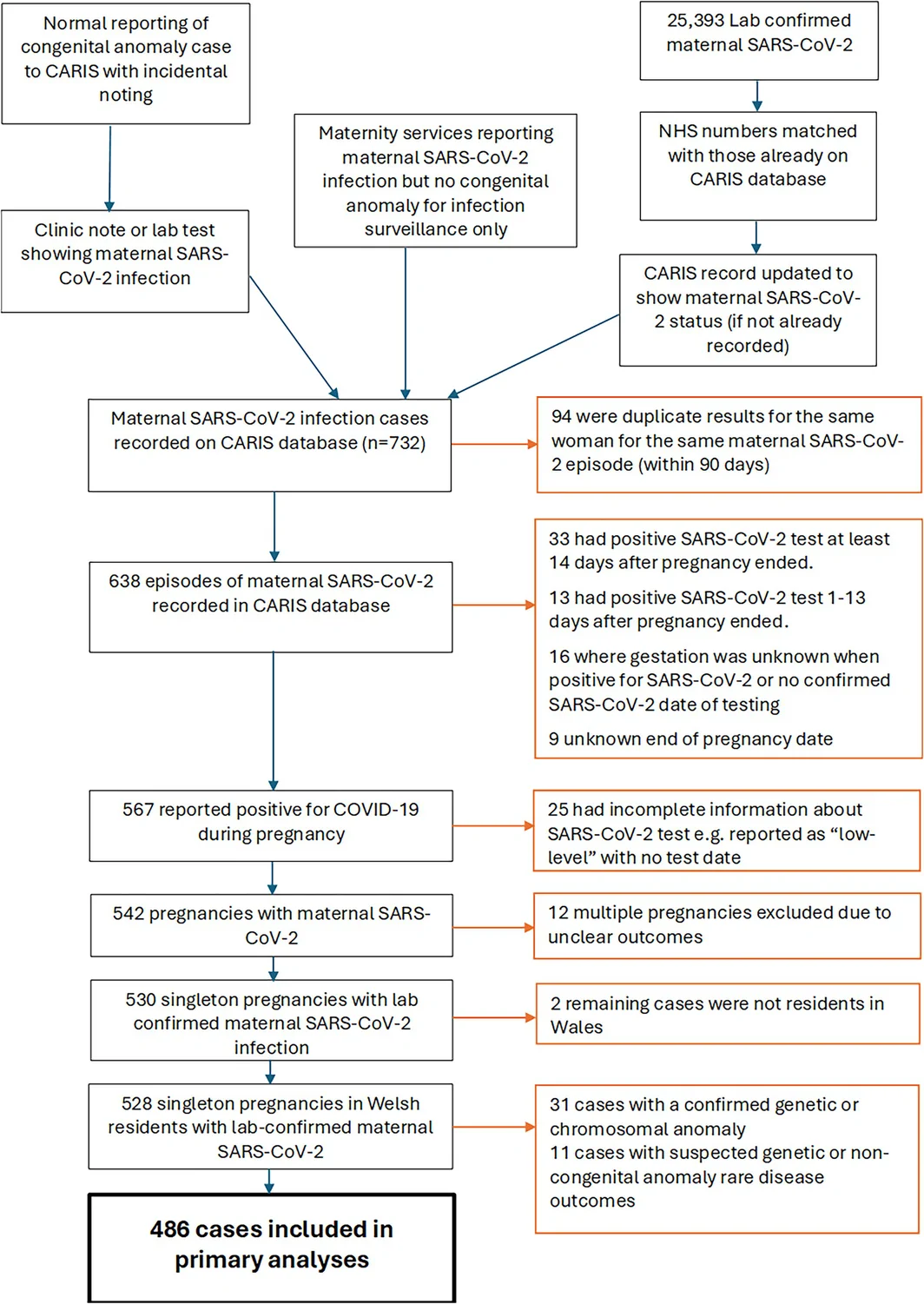
Maternal SARS‐CoV‐2 infection cohort flowchart.

The number of anomalies in each subgroup in the cohort group was compared to those in the Welsh population between 2009 and 2024. Subgroups were grouped by the same CARIS and EUROCAT guidance as the prevalence comparison analysis described previously using the same exclusion criteria as the CARIS official statistics (CARIS 2025).

The total number of anomalies per subgroup in the SARS‐CoV‐2 cohort was calculated and then the proportions of anomalies in the SARS‐CoV‐2 cohort were compared to the proportions reported in the statistics for Wales (2009–2024) using the Newcombe–Wilson comparison method to provide a risk difference per 10,000 cases with 95% confidence intervals. A Holm–Bonferroni adjustment was used to control the family‐wise error rate (FWER) in multiple subgroup analysis as it is less strict than Bonferroni and therefore provides a higher statistical power for Newcombe–Wilson tests (GeeksforGeeks 2025; Team MA 2026).

Statistical analyses for pre‐pandemic and pandemic years comparison and cohort comparison were completed using R Studio version 4.5.0. Maternal demographic analyses were completed using SPSS.

### 2.5 Ethical Approval and Information Governance

Formal medical ethics committee approval was not required for this work. The collection, analyses, and reporting of aggregate and anonymized congenital anomaly data is a statutory function of the Public Health Wales NHS Trust surveillance registries. Collection of COVID‐19 specific data was approved in Wales as part of the pandemic response for protecting the health of the public. Data were managed in accordance with Public Health Wales information governance practices and data protection law including the General Data Protection Regulation 2018. Data with small numbers (aggregate counts of < 5) were suppressed to reduce the risk of individual identification.

## 3 Results

### 3.1 Pre‐Pandemic and Pandemic Comparison of Population‐Level Data

The mean number of congenital anomalies reported by CARIS per year between 2009 and 2019 was 1529 (range 1098–1782), with a general decreasing trend over time (CARIS 2025). The number of anomalies reported in 2020, 2021, and 2022 was lower at 1030, 1154, and 960, respectively. Prevalence of all congenital anomaly cases in Wales reduced during the pandemic (mean 365 per 10,000 in 2020–22 vs. 454 per 10,000 in 2009–2019), demonstrating there was no evidence of a large population‐level increase in the prevalence of congenital anomalies since the beginning of the COVID‐19 pandemic in January 2020 (CARIS 2025). The prevalence of each anomaly subgroup in the two time periods (Figure 2) shows that congenital heart defects are the most common type of congenital anomaly in Wales (mean of 156.9 per 10,000 births pre‐pandemic and a mean of 128.1 per 10,000 births during the pandemic).

**FIGURE 2:**
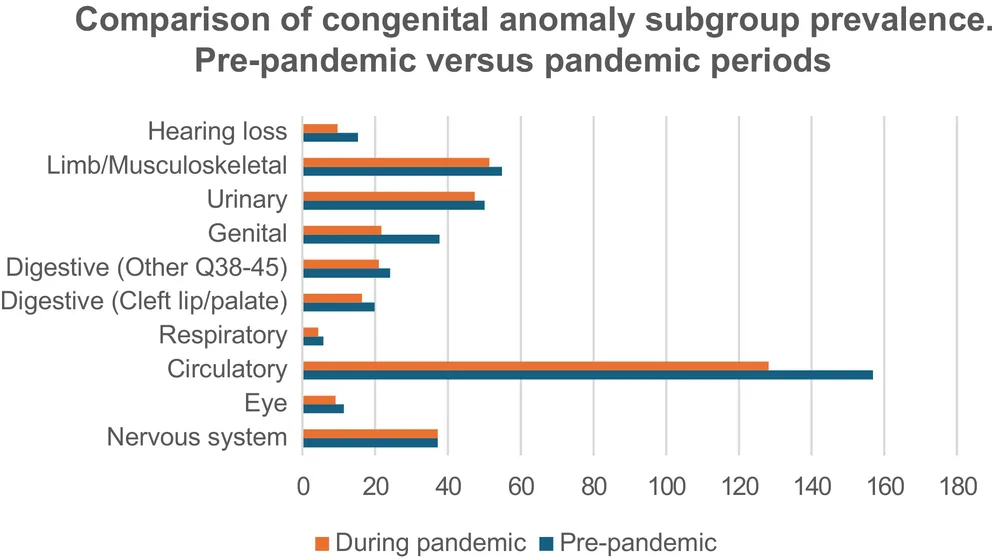
Pre‐pandemic and pandemic comparison of population‐level data.

Congenital anomaly subgroup data from the pandemic years were compared with pre‐pandemic data (Table 1). The only difference noted was an increased prevalence of abdominal wall defects (adjusted *p*‐value of 0.0043).

**TABLE 1:** Prevalence of congenital anomaly subgroups per 10,000 births (2009–2019 vs. 2020–2022) with Benjamini–Hochberg (BH) adjusted *p* value.

Anomaly subgroup	Prevalence 2009–2019	Prevalence 2020–2022	Difference	*z* score	*p* (one tailed)	*p* (BH adjusted)
Nervous system	37.20	37.17	−0.03	−0.0139	0.5060	1.0000
Eye	11.30	9.06	−2.26	−1.8100	0.9650	1.0000
Circulatory	157.00	128.00	−28.80	−6.2200	1.0000	1.0000
Respiratory	5.75	4.30	−1.46	−1.6500	0.9500	1.0000
Oro‐facial clefts	19.70	16.40	−3.37	−2.0400	0.9790	1.0000
Gastri‐intestinal	24.00	21.00	−3.02	−1.6500	0.9500	1.0000
Abdominal wall defects	9.81	13.90	4.13	3.3600	**0.0004**	**0.0043**
Genital	37.70	21.60	−16.10	−7.2300	1.0000	1.0000
Urinary	50.10	47.40	−2.70	−1.0100	0.8450	1.0000
Limb/musculoskeletal	54.80	51.30	−3.48	−1.2500	0.8950	1.0000
Hearing loss	15.20	9.64	−5.57	−3.9100	1.0000	1.0000

*Note:* Bold indicates statsitical significance at the 0.05 level.

Exploration of incidence of congenital anomalies by category over time demonstrated a stable trend. Abdominal wall defects are the only category where cases from 2020 onwards exceeded previously recorded levels since 2009. The mean number of abdominal wall defect cases reported in Wales rose from 33 per year (range 21–39), at a rate of 9.8 per 10,000 births between 2009 and 2019 to 40 cases per year (range 39–41), at a rate of 13.9 per 10,000 births in 2020–2022. This was largely attributed to an increase in omphalocele where the mean between 2009 and 2019 was 15.6 per year (range 10–21), at a rate of 5.0 per 10,000 births, increasing to a mean of 26.3 cases in 2020–2022 (range 25–28), at a rate of 9.2 per 10,000 births.

### 3.2 SARS‐CoV‐2 Infection Cohort Data

The cohort of 486 women with maternal SARS‐CoV‐2 infection consisted mostly of White British women (65%) under the age of 35 years (76.3%), with almost a quarter (23.1%) living in the most deprived areas of Wales.

Timing of SARS‐CoV‐2 infection by trimester of pregnancy for the cohort was 101 (20.8%) during the first trimester of pregnancy, 177 (36.4%) during the second trimester and 208 (42.8%) during the third trimester. The proportion of women positive for SARS‐CoV‐2 and a fetus/baby with a congenital anomaly were similar in the first and second trimesters (40.6% and 41.2% respectively) and lower in the third trimester (27.4%).

Overall, 305/486 (62.8%) had no anomaly reported at birth/end of pregnancy. This included 110 cases where an anomaly had been suspected at some point but had resolved by birth, and 195 where no anomaly was suspected at any point but were prospectively registered with CARIS for SARS‐CoV‐2 surveillance. A total of 325 anomalies were reported in this cohort with a mean of 1.8 anomalies per child.

Congenital heart defects were the most frequently reported anomaly in the SARS‐CoV‐2 cohort (Table 2). The prevalence of congenital heart defects and urinary anomalies was statistically significantly higher in the SARS‐CoV‐2 cohort compared with Wales population level rates (Table 2, Figure 3).

**TABLE 2:** Comparison of congenital anomaly subgroup frequency of maternal SARS‐CoV‐2 infection cohort against population‐level frequency in Wales (2009–2024) using Newcombe–Wilson confidence intervals with Holm–Bonferroni adjustment.

Analysis of 325 anomalies versus 21,599 anomalies—per 10,000|per‐comparison alpha = 0.004167												
Anomaly subgroup	No. cases in cohort	Total subgroup anomalies in cohort	No. cases in Wales (2009–2024)	Total subgroup anomalies in Wales (2009–2024)	Prevalence of anomaly subgroup in cohort per 10,000	Prevalance of anomaly subgroup in Wales (2009–2024) per 10,000	Risk difference per 10,000 births	Lower RD per 10,000 births	Upper RD per 10,000 births	*p*	*p* Holm–Bonferroni adjusted	Confidence interval significant
Nervous system	36	325	1873	21,599	1107.692308	867.169776	240.522531	−218.244774	884.412619	0.126888	0.507553	FALSE
Eye	6	325	528	21,599	184.615385	244.455762	−59.840377	−214.562539	324.173580	0.487304	0.974608	FALSE
Congenital heart defects	99	325	4832	21,599	3046.153846	2237.140608	809.013238	57.588223	1652.852522	0.000526	0.005260	**TRUE**
Respiratory	< 5	325	266	21,599	92.307692	123.153850	−30.846157	−125.614027	297.586413	0.801947	0.974608	FALSE
Oro‐facial clefts	9	325	955	21,599	276.923077	442.150100	−165.227023	−371.984684	266.506465	0.149302	0.507553	FALSE
Gastrointestinal	26	325	1169	21,599	800.000000	541.228761	258.771239	−118.934745	836.234789	0.041383	0.289678	FALSE
Abdominal wall defects	13	325	530	21,599	400.000000	245.381731	154.618269	−90.214178	618.328806	0.075046	0.375228	FALSE
Genital	14	325	1647	21,599	430.769231	762.535303	−331.766072	−608.592496	162.836110	0.024869	0.198950	FALSE
Urinary	65	325	2496	21,599	2000.000000	1155.609056	844.390944	227.591683	1603.382609	0.000003	0.000028	**TRUE**
Limb/musculoskeletal	34	325	3590	21,599	1046.153846	1662.113987	−615.960141	−1078.874830	35.645352	0.003005	0.027045	FALSE
Hearing loss	< 5	325	658	21,599	123.076923	304.643734	−181.566810	−306.698207	175.876782	0.057623	0.345736	FALSE

*Note:* Subgroups less than five were suppressed to reduce the risk of patient identification. Bold indicates statsitical significance at the 0.05 level.

**FIGURE 3:**
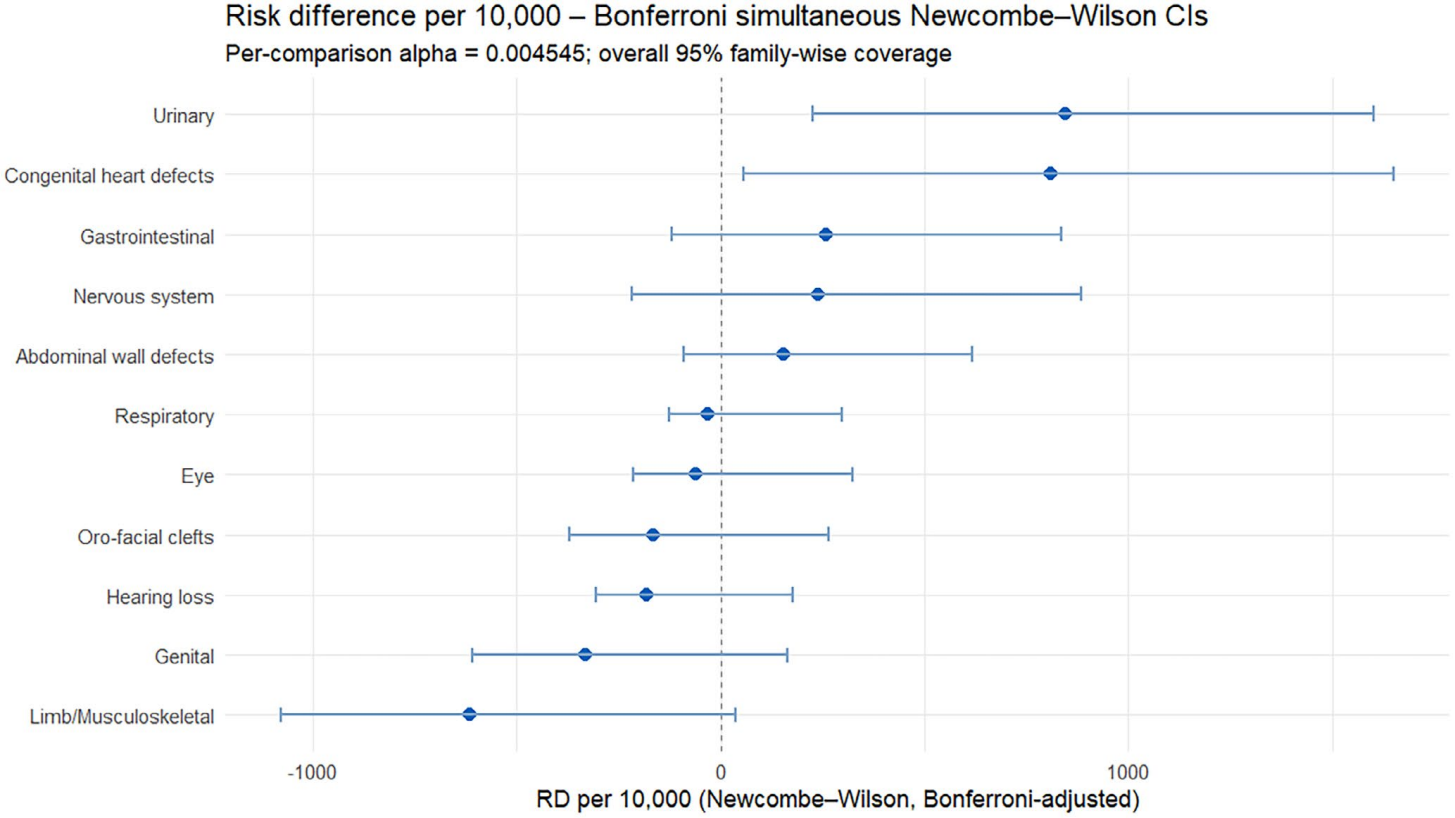
Confidence intervals of the risk difference between maternal SARS‐CoV‐2 infection cohort anomaly subgroups and population‐level congenital anomaly data in Wales (2009–2024).

Omphalocele was not prevalent in the SARS‐CoV‐2 cohort, with less than five cases reported (data suppressed), and infection occurring across different trimesters. The prevalence of abdominal wall defects was not significantly different from the population‐level data (Table 2).

## 4 Discussion

Our findings were in keeping with literature on COVID‐19 and congenital anomalies, with no general association identified between SARS‐CoV‐2 infection and population‐level incidence of congenital anomalies (Hernández‐Díaz et al. 2022; Qu et al. 2025; European Commission, n.d.‐b; Heidarzadeh et al. 2022). Whilst several studies suggest theoretical mechanisms whereby SARS‐CoV‐2 may cause congenital anomalies (Khan et al. 2020; Rizzi et al. 2020), there was limited evidence in practice. Statistical analysis on population‐level data from Wales confirmed that the prevalence of total congenital anomalies had not increased since the start of the COVID‐19 pandemic. The declining birth rate in England and Wales (Population Life Events team 2026) would likely mean a reduction in total cases of congenital anomaly that should stabilize when assessing by birth rate. Health services in Wales, including the CARIS registry, were largely disrupted due to the pandemic, which may have contributed to reduced reporting of congenital anomalies. Congenital anomaly registries often experience a lag of around 18–24 months due to delays in diagnosis or reporting, and the impact of the pandemic may have caused further disruption (Broughan et al. 2024). The lag in reporting from the pandemic years should have stabilized due to time elapsed; however, detection, diagnosis, and reporting of congenital anomalies can vary greatly across subgroups (Segovia Chacón et al. 2023).

We analyzed eleven congenital anomaly subgroups to investigate for any organ specific congenital anomaly prevalence changes. There was a significant increase in the prevalence of the abdominal wall defect subgroup, driven by a rise in omphalocele cases. However, our SARS‐CoV‐2 cohort showed no clear association between infection and omphalocele prevalence. Omphalocele prevalence has been steadily increasing across Europe, but the rise began in 2016 (European Commission, n.d.‐b), so is likely due to other factors rather than maternal SARS‐CoV‐2 infection. This has also been observed in population‐based cohort studies in Scotland and Nordic countries where individual anomaly case numbers were too small to analyze statistically, but there was no evidence of an association between maternal SARS‐CoV‐2 infection when compared as subgroups (Calvert et al. 2023; Magnus et al. 2024).

We did not analyze individual anomalies as we had not identified obvious rises in prevalence of one singular anomaly, and case numbers for many would be too low for meaningful analysis in Wales. Therefore, registries must remain vigilant when monitoring trends or clusters in individual anomalies.

Congenital heart defects were the most frequently reported anomaly subgroup in our SARS‐CoV‐2 cohort (*n* = 99), in‐keeping with population‐level data in Wales from 2009 to 2019 (CARIS 2025). The SARS‐CoV‐2 cohort had a significant increase in prevalence of urinary and cardiac conditions compared to population‐level prevalence. However, subgroup analysis did not reveal an increase in prevalence of urinary or circulatory anomalies in 2020–2022 at population‐level compared with the pre‐pandemic period.

Excluding abdominal wall defects, no association was found between any of the congenital anomaly subgroups in the Q chapter of ICD‐10 at population level in the pre and during pandemic comparison. This was in keeping with findings from other countries (Mostafa et al. 2022; Senthil et al. 2024; Tanyeri Toker et al. 2023). Sensorineural hearing loss was included as CARIS routinely collects this data in Wales, and other infections acquired during pregnancy such as cytomegalovirus have been associated with sensorineural hearing loss. We did not identify an increase in the prevalence of sensorineural hearing loss at population level or within our SARS‐CoV‐2 cohort, matching existing global research (Ma et al. 2021).

Data analysis according to trimester of maternal SARS‐CoV‐2 infection added an additional reassurance to this work. The rationale for this analysis was that teratogens have shown to have the greatest impact when maternal infection occurs during the first trimester. Where anomalies were detected, there were no clear links between the timing of SARS‐CoV‐2 in pregnancy and categories of congenital anomaly by body system. However, it must be acknowledged that there was a likely bias towards recruitment of mothers with SARS‐CoV‐2 infection in the third trimester due to a higher likelihood of opportunistic testing at hospital admission (noting this data was collected prior to the availability of widespread community testing).

Given the sample size constraints in this analysis, we recommend continued surveillance as, though our analysis is reassuring, it is not yet possible to fully exclude a pandemic impact on subgroups or singular anomalies.

## 5 Strengths and Limitations of This Study

This study uses population‐level surveillance data from a national congenital anomaly register that has been reliably collecting data since 1998. Pregnancies are monitored by obstetricians and gynecologists, whereas infections are monitored by virologists and infectious disease specialists. Congenital anomaly surveillance using information from both maternal care and laboratories brings intradepartmental collaboration that enhances wider knowledge of infectious disease in pregnancy. Triangulation has been used to enhance the credibility and validity of the findings, in both collecting and analyzing data. Multiple testing of congenital anomaly subgroups has the potential to produce Type I errors if multiple comparisons are made. This was mitigated with appropriate correction methods.

This study only included data on laboratory confirmed cases, noting that widespread sampling was not available in the early stages of the pandemic and that lateral flow tests were only available in the later stages of the pandemic. The cohort mostly comprised pregnancies affected by congenital anomalies and maternal SARS‐CoV‐2 infection and was not representative of all pregnancies where infection occurred. Therefore, it remains unclear how representative the study cohort was to all women with SARS‐CoV‐2 infection during pregnancy in Wales at population level. Many women infected with SARS‐CoV‐2 during pregnancy would not have been detected; around half of women with SARS‐CoV‐2 infection during pregnancy worldwide were asymptomatic (National Community Child Health Database 2025). Information on SARS‐CoV‐2 testing (including indication), severity, treatment and outcome of SARS‐CoV‐2 infection was not available, and we did not have the vaccination status of the mother. Much of the maternal factors and characteristics of the 486 women in the SARS‐CoV‐2 cohort such as smoking status, body mass index, diabetes and substance misuse were unknown and limited further exploration. This is a common problem in capturing data for surveillance registries.

The observed increase in omphalocele prevalence that began before the pandemic makes analysis of the impact of maternal SARS‐CoV‐2 infection difficult regarding this specific anomaly. There may also be recruitment bias in the cohort group in which mothers were tested, recorded on health records and notified to CARIS. We acknowledge the limitation of observational data and suggest that this work not be interpreted in isolation; rather, that it adds to the growing body of evidence in the literature.

## 6 Conclusion

There was no clear evidence of a population‐level association between congenital anomalies and maternal SARS‐CoV‐2 infection in Wales. Potential association with specific congenital anomalies cannot be excluded. The Europe‐wide increase in omphalocele prevalence observed is not thought to be due to the COVID‐19 pandemic. National surveillance registries should remain vigilant. Due to the rarity of congenital anomalies, prospective data collection and further analyses at international level are recommended to ensure association between SARS‐CoV‐2 infection and specific anomalies do not emerge.

This study adds evidence to the global body of literature on congenital anomalies and the impact of the pandemic by confirming that Wales has not observed a rise in congenital anomaly prevalence since early 2020.

## Funding

The work was supported by the employing organisations as part of usual service work. RMJ is funded by the LifeArc Centre for the Acceleration of Rare Disease Trials.

## Ethics Statement

The work was completed as part of usual service work for the employing organization and was managed in line with organizational policies. No direct ethical approval was sought for this work.

## Conflicts of Interest

Authors (named) are employed by the employing organization, which supported this work through usual service delivery.

## Supporting information


**Data S1:** Number and rate per 10,000 population of reported congenital anomalies in Wales, per year, from 2009 to 2024, reported by the Congenital Anomaly Register and Information Service (CARIS).

## Data Availability

The data underlying this article will be shared on reasonable request to the corresponding author.
