# Supplementary material for: Is SARS‐CoV‐2 Infection (COVID‐19) Associated With Congenital Anomalies? A Population Study of Congenital Anomaly Surveillance Data and Maternal SARS‐CoV‐2 Infection in Wales
Source: Birth Defects Res. 2026 Oct 30;118(11):e70122. doi: 10.1002/bdr2.70122 (PMC13722829; doi:10.1002/bdr2.70122)
Supplement: Supplementary file 1 [file BDR2-118-e70122-s001.docx]

Supplementary file: Number and rate per 10,000 population of reported congenital anomalies in Wales, per year, from 2009-2024, reported by the Congenital Anomaly Register and

**NUMBER OF CASES**																	
**Anomaly**	**ICD-10 Codes**	**2009**	**2010**	**2011**	**2012**	**2013**	**2014**	**2015**	**2016**	**2017**	**2018**	**2019**	**2020**	**2021**	**2022**	**2023**	**2024[a]**
**All including minor anomalies**		1777	1782	1754	1704	1569	1555	1426	1337	1381	1434	1098	1030	1154	960	893	745
**Nervous system anomalies**	Q00 - Q07, Q87.03	136	139	125	129	143	120	129	117	103	119	117	100	107	113	99	77
**Congenital heart defects**	Q20 - Q26	421	405	361	349	347	323	293	320	314	299	263	236	250	218	216	217
**Respiratory anomalies**	Q30.0, Q32 - Q34	24	21	28	28	16	17	15	18	11	19	16	12	15	10	11	5
**Oro-facial clefts**	Q35 - Q37	79	65	74	73	76	59	69	46	71	79	40	60	43	38	48	35
**Gastro-intestinal anomalies**	Q38 - Q45, Q79.0	105	77	85	95	89	87	78	64	83	62	65	59	72	50	50	48
Abdominal wall defects	*Q79.2, Q79.3, Q79.5*	*38*	*39*	*39*	*34*	*38*	*34*	*32*	*28*	*33*	*27*	*21*	*39*	*41*	*40*	*25*	*22*
Omphalocele	*Q79.2*	*17*	*16*	*17*	*15*	*14*	*19*	*21*	*13*	*16*	*14*	*10*	***26***	***28***	***25***	*11*	*11*
**Kidney and urinary tract**	Q60 - Q64, Q79.4	160	173	174	183	149	190	156	164	155	186	164	144	133	131	130	104
**Genital anomalies**	Q50 - Q52, Q54 - Q56	127	133	152	148	129	132	114	109	120	140	91	54	66	66	34	32
**Limb anomalies**	Q65 - Q74	194	191	184	194	169	182	205	194	178	198	140	139	175	128	122	97
**Sensorineural hearing loss**	H90.3 - H90.9	68	74	52	46	34	63	49	46	49	43	39	31	28	24	8	4
**Eye anomalies**	Q10 - Q15	51	45	36	44	42	32	41	33	32	35	28	26	29	23	17	14
**RATE PER 10,000**																	
**Anomaly**		**2009**	**2010**	**2011**	**2012**	**2013**	**2014**	**2015**	**2016**	**2017**	**2018**	**2019**	**2020**	**2021**	**2022**	**2023**	**2024[a]**
**All including minor anomalies**		506	493.2	490.3	481.1	462.9	461.1	426.5	404	427.2	456.6	368	357.9	399.2	337.9	325	276.3
**Nervous system anomalies**	Q00 - Q07, Q87.03	38.7	38.5	34.9	36.4	42.2	35.6	38.6	35.4	31.9	37.9	39.2	34.7	37	39.8	36	28.6
**Congenital heart defects**	Q20 - Q26	119.9	112.1	100.9	98.5	102.4	95.8	87.6	96.7	97.1	95.2	88.2	82	86.5	76.7	78.6	80.5
**Respiratory anomalies**	Q30.0, Q32 - Q34	6.8	5.8	7.8	7.9	4.7	5	4.5	5.4	3.4	6	5.4	4.2	5.2	3.5	4	1.9
**Oro-facial clefts**	Q35 - Q37	22.5	18	20.7	20.6	22.4	17.5	20.6	13.9	22	25.2	13.4	20.8	14.9	13.4	17.5	13
**Gastro-intestinal anomalies**	Q38 - Q45, Q79.0	29.9	21.3	23.8	26.8	26.3	25.8	23.3	19.3	25.7	19.7	21.8	20.5	24.9	17.6	18.2	17.8
Abdominal wall defects	*Q79.2, Q79.3, Q79.5*	*10.8*	*10.8*	*10.9*	*9.6*	*11.2*	*10.1*	*9.6*	*8.5*	*10.2*	*8.6*	*7*	*13.6*	*14.2*	*14.1*	*9.1*	*8.2*
Omphalocele	*Q79.2*	*4.8*	*4.4*	*4.8*	*4.2*	*4.1*	*5.6*	*6.3*	*3.9*	*4.9*	*4.5*	*3.4*	***9***	***9.7***	***8.8***	*4*	*4.1*
**Kidney and urinary tract**	Q60 - Q64, Q79.4	45.6	47.9	48.6	51.7	44	56.3	46.7	49.6	47.9	59.2	55	50	46	46.1	47.3	38.6
**Genital anomalies**	Q50 - Q52, Q54 - Q56	36.2	36.8	42.5	41.8	38.1	39.1	34.1	32.9	37.1	44.6	30.5	18.8	22.8	23.2	12.4	11.9
**Limb anomalies**	Q65 - Q74	55.2	52.9	51.4	54.8	49.9	54	61.3	58.6	55.1	63	46.9	48.3	60.5	45.1	44.4	36
**Eye anomalies**	H90.3 - H90.9	14.5	12.5	10.1	12.4	12.4	9.5	12.3	10	9.9	11.1	9.4	9	10	8.1	6.2	5.2
**Sensorineural hearing loss**	Q10 - Q15	19.4	20.5	14.5	13	10	18.7	14.7	13.9	15.2	13.7	13.1	10.8	9.7	8.4	2.9	1.5

[a] May be subject to lag in reporting
